# Periductal iron-corrected T1 is a predictor of adverse outcomes in large-duct primary sclerosing cholangitis

**DOI:** 10.1186/s12880-026-02242-1

**Published:** 2026-03-04

**Authors:** Emmanuel A. Selvaraj, Ahmed Ba-Ssalamah, Sarah Poetter-Lang, Bruno Paun, Gerard R. Ridgway, Cayden Beyer, Anneli Andersson, Sir Michael Brady, Michele Pansini, Emma L. Culver, Adam Bailey, Michael Pavlides DPhil

**Affiliations:** 1 Oxford Centre for Clinical Magnetic Resonance Research (OCMR), Radcliffe Department of Medicine, University of Oxford, Oxford, UK; 2 Translational Gastroenterology and Liver Unit, Nuffield Department of Medicine, University of Oxford, Oxford, UK; 3 NIHR Oxford Biomedical Research Centre, University of Oxford and Oxford University Hospitals NHS Foundation Trust, Oxford, UK; 4 South Warwickshire University Hospital Foundation Trust, Warwick, UK; 5 Department of Biomedical Imaging and Image- guided Therapy, Medical University of Vienna, General Hospital of Vienna (AKH), Vienna, Austria; 6 Perspectum Ltd., Oxford, UK; 7 Oxford Brain Diagnostics Ltd., Oxford, UK; 8 Medical Sciences Division, Department of Oncology, University of Oxford, Oxford, UK; 9 Department of Radiology, Clinica Di Radiologia EOC, Istituto Di Imaging Della Svizzera Italiana, Lugano, Switzerland

**Keywords:** Bile duct, Cholestasis, Liver fibrosis, MRCP, Magnetic resonance imaging, cT1, PSC, Peribiliary inflammation

## Abstract

**Background:**

Concentric periductal fibrosis, commonly referred to as “onion skin” fibrosis, is a histological hallmark of primary sclerosing cholangitis (PSC) and associated with the development of biliary-type cirrhosis and end-stage liver disease. Our aim was to investigate whether non-contrast enhanced quantitative MRI can provide a noninvasive biomarker of periductal fibro-inflammation in large-duct PSC.

**Methods:**

This prospective cohort study included adults with diagnosis of large-duct PSC, at baseline and 1-year follow-up, and healthy volunteers (HV) at baseline. MRCP + and LiverMultiScan (both Perspectum, UK) were used to align 3D biliary tree models with axial iron-corrected T1 (cT1) maps to quantify cT1 around the bile ducts (periductal-cT1; Pd-cT1). Regions of interest (ROIs) were defined as rings of tissue at increasing distance from the bile duct (1.5–3.5 mm (1), 3.5–5.5 mm (2), 5.5–7.5 mm (3) or 7.5–9.5 mm (4), whole liver (5)). Pd-cT1 was compared between high-risk or low-risk groups using existing markers of risk in PSC and Cox proportional hazard model was used for prediction of outcomes by elevated (> 800ms) Pd-cT1.

**Results:**

From 80 recruited participants with PSC, *n* = 72 had available Pd-cT1 at baseline (median age 44 years; 65% male) and *n* = 20 HV were included (median age 35 years; 65% male). In PSC, Pd-cT1 in ROIs 1 and 2 was higher than ROIs 3–4 and whole liver cT1 (*p* < 0.0001) at baseline and follow-up, while there were no differences between ROIs in HV. Participants with high risk stratified by liver stiffness (LS) > 9.6 kPa, enhanced liver fibrosis (ELF) test > 9.8 and Amsterdam-Oxford Model (AOM) > 2 had significantly higher Pd-cT1 than those with low risk classification. In a median follow-up of 49 months (range: 14–66), elevated baseline Pd-cT1 could predict adverse events (cholangitis, hepatic decompensation, liver transplantation and all-cause mortality) in patients with large-duct PSC.

**Conclusions:**

Pd-cT1, a novel quantitative MRI method combining biliary and parenchymal technologies, detected periductal liver abnormalities in PSC and was associated with increased risk of clinical outcomes. This work proposes a non-invasive, objective biomarker of fibro-inflammation in the periductal parenchyma in PSC which, once validated, may have utility as a monitoring biomarker in clinical trials for large-duct PSC.

**Supplementary Information:**

The online version contains supplementary material available at 10.1186/s12880-026-02242-1.

## Background

Primary sclerosing cholangitis (PSC) is a chronic cholangiopathy marked by inflammation and fibrosis of the intra- and/or extrahepatic bile ducts, leading to progressive ductal strictures, cholestasis, and eventually biliary-type cirrhosis [1]. A hallmark histological feature of PSC is concentric periductal fibrosis, also known as “onion-skin” fibrosis, resulting from chronic peribiliary inflammation and stromal expansion [2, 3]. This fibro-obliterative process begins in the portal tracts, progresses along a portal-to-portal pattern, and ultimately disrupts normal lobular architecture [4]. While liver biopsy can detect these changes, its use is limited by sampling variability and procedural risk in a disease with patchy distribution [5].

Peribiliary hyperenhancement, seen with contrast-enhanced magnetic resonance imaging (MRI), is thought to reflect this enhanced disease activity and fibrosis in the periductal region of the liver parenchyma in PSC [6, 7]. Peribiliary hyperenhancement has been shown to correlate with the revised Mayo Risk Score, associate with higher segmental liver stiffness on magnetic resonance elastography (MRE) [8] and associate with composite transplant-free survival [7]. However, the need for contrast administration, perfectly timed acquisition and manual measurements makes the method challenging in practice. Furthermore, inter-observer variability in standard MRI interpretation in PSC, including magnetic resonance cholangiopancreatography (MRCP) is high, even among experts [9–11]. Standardised quantitative multiparametric MRI methods have shown promise in overcoming these limitations by robust quantification of biliary tree anatomy and markers of fibro-inflammation in the liver parenchyma [12, 13].

Iron-corrected T1 (cT1), derived from multiparametric MRI of liver T1 and T2*, to account for the confounding effect of liver iron on the T1 measurement [14, show excellent repeatability and reproducibility across scanner vendors and field strengths [13]. cT1 correlates with liver fibrosis on biopsy [15, 16] and elevated cT1 indicate presence of fibro-inflammatory disease activity, such as steatohepatitis or active autoimmune hepatitis [17–19]. Furthermore, liver cT1 can predict liver- and cardiovascular-related outcomes [20–22, and is used to assess changes following interventions in steatotic and autoimmune liver disease [19, 23]. Quantitative MRCP generate parametric 3D models of the biliary tree and derive biliary metrics with high repeatability and reproducibility [12, 24]. Several of these biliary metrics have been shown to associate with disease severity and clinical outcomes in PSC [25–27]. By aligning the MR acquisitions and co-registering the models of quantitative MRCP and cT1 maps, periductal cT1 metrics can be quantified.

The aim of this study was to develop a method for measuring cT1 in the liver parenchyma immediately surrounding bile ducts (periductal cT1 [Pd-cT1]) and to evaluate the clinical significance of this metric in risk stratification of large-duct PSC.

## Methods

### Study design

This was a prospective, single-centre, longitudinal study at a tertiary non-transplant centre (Oxford University Hospitals National Health Service Foundation Trust). The study protocol conformed to the ethical guidelines of the 1975 Declaration of Helsinki and the principles of good clinical practice. All participants provided written informed consent. Ethical approval was granted by the National Research Ethics Service, and by the local Research and Development Department. Study assessments were conducted for participants with PSC and healthy volunteers at baseline (visit 1) and repeated in those with PSC after 12 months (visit 2).

### Participant selection

#### PSC

Adults (≥ 18 years) with a known diagnosis of large-duct PSC, defined according to the European Association for the Study of the Liver (EASL) guidelines [28] as the association of chronic cholestasis, typical features on MRCP and no cause of secondary sclerosing cholangitis, were recruited. Participants were excluded if they had contraindications to MRI scanning, other proven or suspected co-existing cholangiopathy (e.g. cholangiopathy related to recreational ketamine use), small-duct PSC, overlap with primary biliary cholangitis, previous choledochojejunostomy, previous liver transplant, cholangiocarcinoma, hepatocellular carcinoma, cirrhosis decompensation at the time of inclusion, clinical or laboratory evidence of a liver diagnosis other than PSC or consumed more alcohol than the current limit recommended by the UK Department of Health (14 units/week or 16–24 g/day).

#### Healthy volunteers

Age and sex matched healthy volunteers with normal liver biochemistry, no previous self-reported history of liver or biliary disease or intervention, alcohol consumption within the current recommended limit as above, and body mass index not greater than 25 kg/m^2^, were recruited for reference.

### Clinical and laboratory data

Prior to the MRI, fasting blood samples were taken for alkaline phosphatase (ALP), aspartate aminotransferase (AST), alanine aminotransferase (ALT), gamma-glutamyltransferase (GGT), bilirubin, albumin, platelet count, prothrombin time (PT), and enhanced liver fibrosis (ELF™; Siemens Healthineers, Erlangen, Germany). Transient elastography liver stiffness (LS) was performed using FibroScan^®^ 502 Touch (Echosens, Paris) by trained operators using the automatic probe selection tool on the same day. For a successful measurement, 10 valid readings were required and as per recommended guidelines, unreliable readings were defined as having an interquartile range (IQR)/median ratio > 0.3. Electronic medical records were searched to obtain relevant clinical data (age at diagnosis, presence of inflammatory bowel disease (IBD), medication history, history of variceal bleeding, and distribution of disease) and long-term follow-up of clinical events (incidence of liver adverse events including cholangitis, development of new dominant stricture (DS), the need for endoscopic retrograde cholangiopancreatography (ERCP), hepatic decompensation, liver transplantation, cancer and all-cause mortality).

### Risk classification

Non-invasive surrogate predictive markers of disease severity and progression were used as a reference in this study. The selection of thresholds were literature-based and aimed at selecting those with elevated risk of clinical outcomes, including due to advanced parenchymal fibrosis or elevated prognostic risk scores. Participants with PSC were classified into high-risk or low-risk groups according to the following criteria:

#### Liver fibrosis markers

High-risk PSC was defined as LS by transient elastography of > 9.6kPa [29] or ELF score > 9.8 [30] as these cut-offs correlate with histologically-confirmed advanced fibrosis and clinical outcomes.

#### Prognostic risk models

The Amsterdam-Oxford Model (AOM) predicts long-term risk of PSC-related death and/or liver transplantation [31, and AOM > 2 was defined as high-risk [32]. The revised Mayo Risk Score provides 4-year mortality risk in PSC [33] and in this study a score > 0 was defined as high-risk [7, 34].

#### Serum ALP

Both the cut-off value of 1.5 times the upper limit of normal (xULN) [35] and ≥ 2.2 xULN have shown predictive ability of liver transplantation and survival in PSC [36] and were examined in our study.

#### Qualitative MRCP scores

The Anali score without gadolinium [37, 38] 3–5 was classified as high-risk in this study. The modified Amsterdam intrahepatic stricture severity score (ISSS) ranged from 0 to 4 and 3–4 was classified as high risk in this study [39–41].

#### Qualitative MRCP features

The presence of extrahepatic biliary disease was associated with adverse outcomes in a large UK multicentre PSC research cohort [36] and the same disease distribution classification was used in our study for risk stratification. The presence of dominant strictures [42] was also classified as high risk.

### MRI acquisition

All participants attended a study visit after fasting for at least four hours. T2-weighted 3D MRCP, T1 and T2* mapping as well as a 3D VIBE scans were acquired on a 3T scanner (Magnetom Prisma, Siemens Healthineers, Erlangen, Germany). All sequences were consecutively acquired in the same scan session without moving the participant from the MRI scanner and using respiratory gating. Acquisition parameters are summarised in Table S1.

### Image analysis

#### MRI-MRCP interpretation

Both MRI and MRCP sequences of participants with PSC were read by two expert hepatopancreatobiliary radiologists with a subspecialist interest in PSC (ABS and SPL with 25 and 8 years of experience, respectively). Both radiologists independently scored the components of modified Amsterdam stricture severity scores (Table S2) [41] and Anali score (Table S3) [37]. A dominant stricture was defined as a stricture less than 1.5 mm diameter in the CBD, or less than 1 mm in the left or right main hepatic ducts. This is now updated in the AASLD and EASL guidelines to “high-grade stricture” defined as a biliary stricture on MRI/MRCP with > 75% reduction of duct diameter in the common bile duct or hepatic ducts, with the indication to use “high-grade stricture” when working with MRCP images [28]. Radiologists were blinded to clinical and quantitative MRI-MRCP data.

#### Alignment of axial liver cT1 maps and biliary tree model

**Fig. 1:**
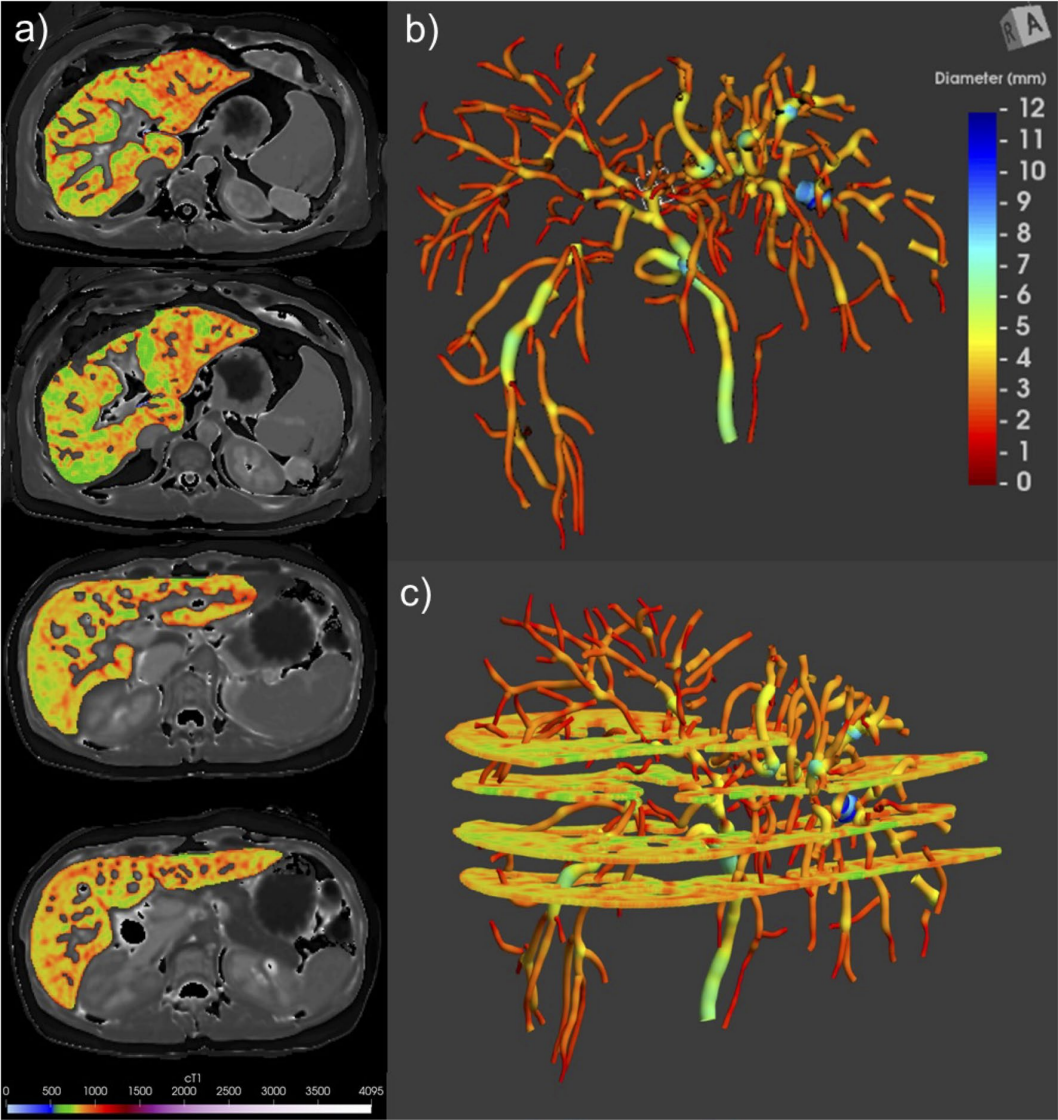
Co-registration of two quantitative MRI techniques for Pd-cT1 quantification in large-duct PSC. (**a**) Four segmented liver axial cT1 maps from Liver*MultiScan* analysis acquired at different levels with prominent blood vessels excluded and liver parenchyma colour-coded to liver cT1 values. (**b**) 3D biliary tree model derived from *MRCP+* analysis, colour-coded according to duct diameter size and orientation in geometric space depicted in the top right corner. (**c**) Co-registration of both images using MRI acquisition coordinates for the purpose of Pd-cT1 quantification

Anonymised MRI-MRCP data were analysed using MRCP + and LiverMultiScan by trained image analysts blinded to the clinical data. Image analysts have continuous training and assessments of inter-operator agreement, to ensure standardized quantitative output from MRCP + and LiverMultiScan. Quantitative parametric 3D models of the biliary tree and their corresponding four axial cT1 maps of liver parenchyma were derived and aligned using the MRI acquisition coordinates and linear interpolation as shown in Fig. 1. Visual inspections were performed to ensure accurate alignment and scans were excluded if there was gross movement artifact or insufficient imaging quality on either of the sequences. Biliary ducts were considered visible when a high-intensity signal was present on MRCP images and the ducts were successfully segmented by the quantitative reconstruction algorithms; in this cohort, the minimum diameter among all reconstructed ducts was 0.8 mm.

#### Pd-cT1 quantification

**Fig. 2:**
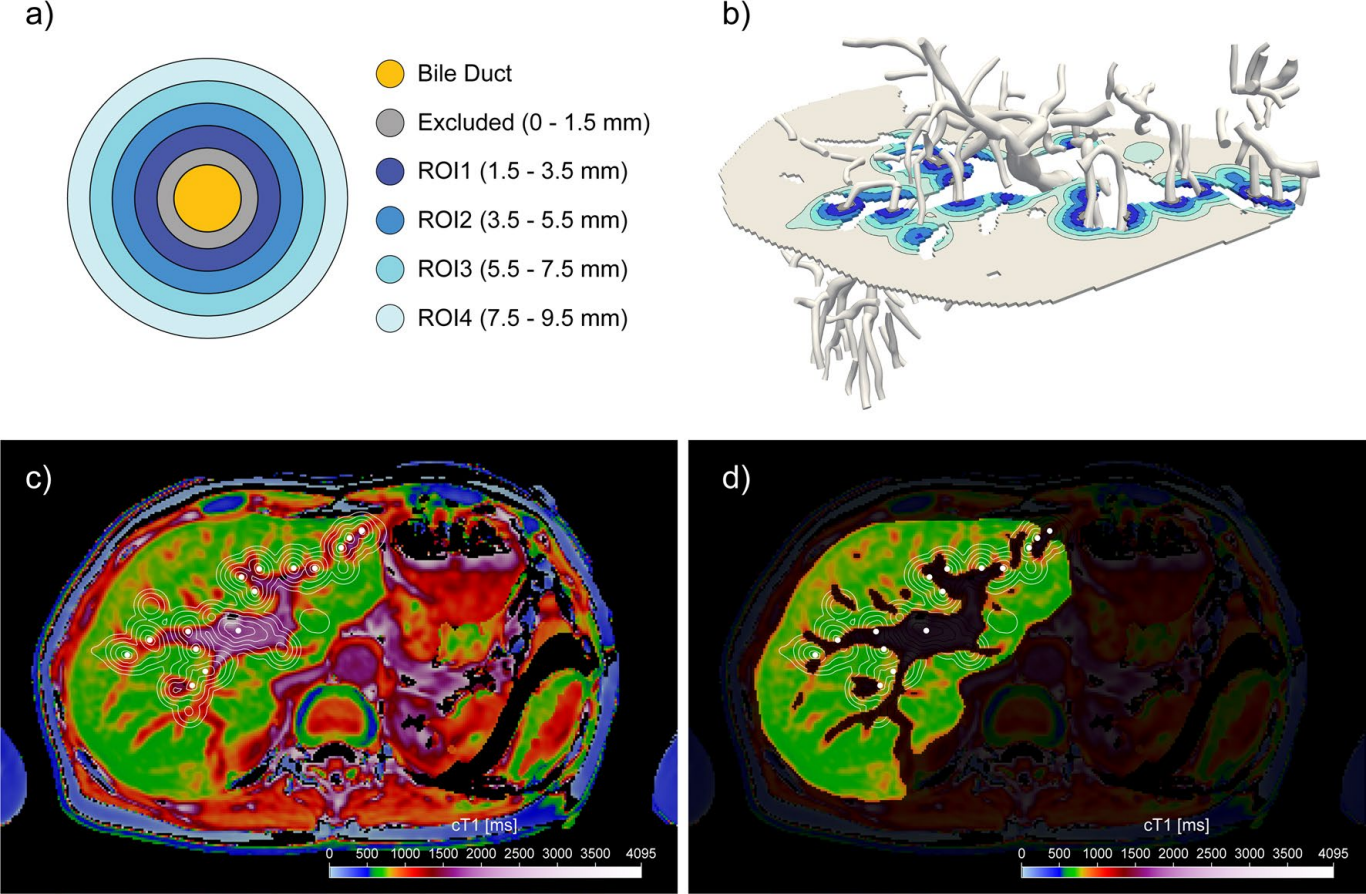
Periductal distance map for a single axial liver slice. **a**) Schematic diagram representing the regions of interest for Pd-cT1 quantification. **b**) 3D representation of defined periductal ROIs on single segmented liver axial slice obtained by 3D distance transform of segmented biliary tree image mask. White mesh represents parametric model of biliary tree and contours lines represent level sets of distance function at 1.5, 3.5, 5.5, 7.5 and 9.5 mm in liver axial plane. (**c**-**d**) 2D representation of co-registered 3D periductal distance map and 2D axial cT1 image. White dots represent intersections of bile duct centrelines with axial cT1 image while contour lines represent level sets of distance map at defined regions of interest (ROI) boundaries. Figure **d**) shows region where Pd-cT1 values were calculated using liver mask to exclude major blood vessels

Pd-cT1 was defined as the mean cT1 in the liver parenchyma within ring shaped regions of interests (ROIs) around the bile duct **(**Fig. 2a**)**. The ring shaped ROIs were generated automatically using co-registered 3D distance map calculated from 3D biliary tree segmentation, then linearly resampled onto cT1 map slices. The ROIs on the cT1 maps were defined by grouping voxels using defined distance ranges from the edge of all the visible bile ducts. ROI 1 was defined as the ring of tissue between 1.5 and 3.5 mm, ROI 2 between 3.5 and 5.5 mm, ROI 3 between 5.5 and 7.5 mm, and ROI 4 between 7.5 and 9.5 mm. For reference, cT1 value of the whole liver was defined as ROI 5. The quantification relied on 3D distance in cT1 maps and biliary tree segmentation masks. Automatic cleaning of potential segmented gastrointestinal contamination in biliary tree masks were performed to follow the corresponding *MRCP+* biliary tree parametric centreline model. Voxels located further than 1.15 times the duct radius from the closest point of corresponding biliary tree duct centreline were excluded. A 15% tolerance in modelled duct radius at each point of a centreline model was used to account for duct deviations from the cylindrical profile. Periductal distance maps were obtained by calculating 3D distance transform of corrected biliary tree mask and was fused onto corresponding co-registered 2D cT1 axial image slices using standard linear interpolation to account for differences in image resolution (Fig. 2b-d**)**. 2D cT1 image slices were further masked using liver mask from LiverMultiScan. The quality criteria for inclusion of Pd-cT1 quantification was a coefficient of determination (R^2^) value of > 98% within the segmented liver portion. The angle at which bile ducts intersected the liver was assessed for partial volume correction as outlined in detail in the Supplementary Methods. Given that the MRCP acquisition voxel size was 1.1 × 1.1 × 1.1 mm, Pd-cT1 measurements below 1.5 mm distance from the bile duct wall were excluded to minimise partial volume effect. A sensitivity assessment in regions where bile ducts traverse through 2D image slices at an oblique angle was performed to assess the partial volume effect. Angles between slice normals and biliary tree duct segments at intersection points were calculated, grouped by < 30°, 30–60° and 60–90° and Pd-cT1 compared, Figure S3.

#### Clinical outcomes

The primary clinical outcome was transplant-free survival (liver transplant or all-cause mortality), with secondary outcomes adding (1) hepatic decompensation and (2) episodes of cholangitis. Elevated cT1 is defined as above the upper limit of normal (800ms) [43, which has shown good discriminatory performance for diagnosis of chronic liver diseases (e.g. MASH and AIH) and significantly higher risk of cardiovascular- and liver-related outcomes in the UK biobank [18, 21, 22, 44].

### Statistical analysis

Descriptive statistics were used to summarise participant characteristics. Normality was assessed using the Shapiro-Wilk test. Results were reported using mean and standard deviation (SD) if parametric distribution or median and interquartile range (IQR) if non-parametric distribution. Associations between continuous variables were tested using the Pearson’s correlation coefficient for parametric variables and Spearman’s rank correlation coefficient for non-parametric variables. Paired comparison between ROIs were performed using a one-way ANOVA with Tukey’s post-hoc correction for multiple comparisons. Receiver operating characteristic curve (ROC) analyses were performed to assess the performance of Pd-cT1 to differentiate between the high-risk and low-risk groups. Cut off values were selected using the Youden index with the corresponding area under the curve (AUC), sensitivity and specificity reported. 2 × 2 mixed-effects ANOVA models were used to examine for potential changes in Pd-cT1 (dependent variable) between the low-risk and high-risk groups (between-subject variable) over the two time points (within-subject variable). The hazard ratio (HR) for prognostic performance of Pd-cT1 to predict adverse events was investigated using the cox proportional hazard model. The data-driven optimal thresholds for predicting primary and secondary outcomes were derived using using maximization of the log-rank statistic (R’s survminer package). Statistical significance was set at *P* < 0.05, and 95% CIs were calculated where appropriate. Analysis was performed using GraphPad Prism version 9.0 for Windows (San Diego, CA), IBM SPSS (v25; IBM Corp., Armonk, NY) and R (version 4.5.1; R Foundation for Statistical Computing, Vienna, Austria).

## Results

### Cohort characteristics

**Fig. 3:**
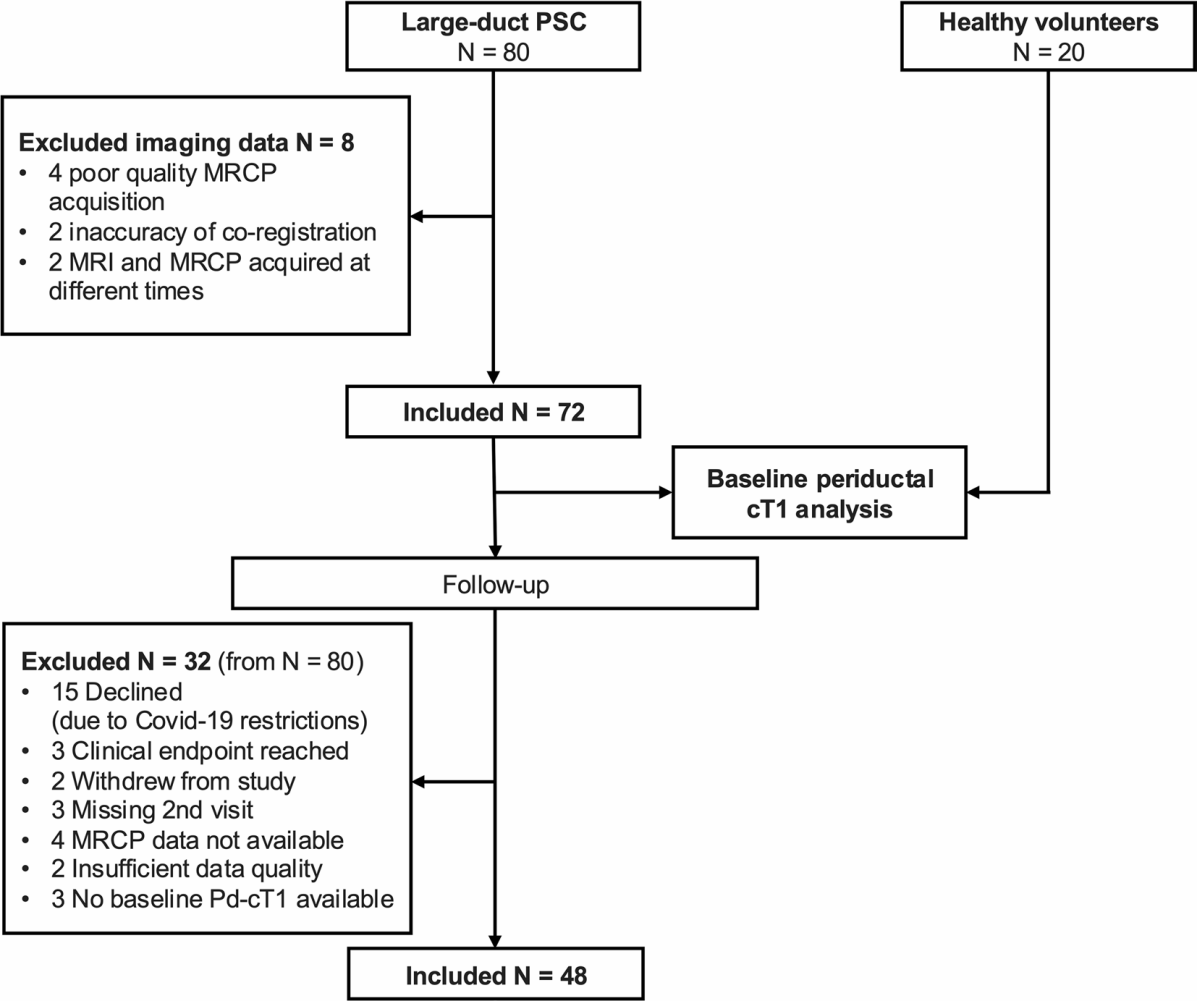
Baseline and follow-up study flow chart

The median age of the *N* = 72 participants with PSC was 44 years (IQR: 25 years) and 65% were male. The median disease duration was 8 years (IQR: 4–12 years), all participants had established PSC during their adult life. 56% of participants were on ursodeoxycholic acid therapy, 76% had concomitant IBD (73% ulcerative colitis) and 56% had both intrahepatic and extrahepatic disease distribution. Further clinical and biochemical parameters for participants with PSC and healthy volunteers are summarised in Table 1 and Table S4, respectively. Pd-cT1 quantification was successful in all healthy volunteers and 72/80 (90%) participants with PSC in the baseline analysis and 48/72 had a paired follow-up result, further detailed in the study flow chart in Fig. 3.

**Table 1:** Participant characteristics at baseline study visits

Large-duct PSC		
	*n* = 72	
**Demographics**		
Male, n (%)	47	(65)
Age (years)	44	(31–56)
**Clinical data**		
PSC duration (years)	8	(4–12)
PSC disease distribution, n (%)		
Intrahepatic + extrahepatic	40	(56)
Intrahepatic only	32	(44)
On UDCA therapy, n (%)	40	(56)
IBD present, n (%)	55	(76)
IBD phenotype, n (%)		
Ulcerative colitis	40	(73)
Crohn’s	8	(15)
Unspecified	7	(13)
**Laboratory parameters**		
Total bilirubin (µmol/l)	13	(10–20)
ALP (IU/l)	152	(102–235)
ALT (IU/l)	42	(27–84)
AST (IU/l)	38	(27–61)
GGT (IU/l)	123	(49–305)
Albumin (g/l)	40	(37–41)
Prothrombin time (s)	10	(10–11)
Platelet count (x 10^9^/l)	256	(191–305)
**Liver fibrosis markers**		
VCTE liver stiffness (kPa)	6.8	(5.3–9.6)
ELF score	9.3	(8.7–9.9)
**Pd-cT1 (ms)**		
Whole Liver cT1	772	(731–800)
ROI 1 (1.5–3.5 mm)	792	(752–825)
ROI 2 (3.5–5.5 mm)	775	(743–811)
ROI 3 (5.5–7.5 mm)	761	(726–804)
ROI 4 (7.5–9.5 mm)	763	(730–801)
**Prognostic risk models**		
Amsterdam-Oxford model score	1.6	(1.3–2.0)
Mayo risk score	0.1	(-0.2–0.4)
**Baseline risk classifications**	**N**	**%**
xULN ALP		
> 1.5x	24	(33)
≤ 1.5x	48	(67)
VCTE liver stiffness (kPa)		
> 9.6	19	(26)
≤ 9.6	53	(74)
ELF score		
> 9.8	20	(28)
≤ 9.8	53	(74)
Amsterdam-Oxford model score		
> 2	22	(31)
≤ 2	50	(69)
Mayo risk score		
> 0	40	(56)
≤ 0	32	(44)
Anali score		
> 2	26	(36)
≤ 2	46	(64)
ISSS		
> 2	36	(50)
≤ 2	36	(50)
Extrahepatic disease		
Yes	40	(56)
No	32	(44)
Dominant stricture		
Yes	24	(33)
No	48	(67)

Continuous variables are expressed as median (interquartile range) and nominal variables as absolute number (percentage). Abbreviations: ALP, alkaline phosphatase; ALT, alanine aminotransferase; AST, aspartate aminotransferase; ELF, enhanced liver fibrosis score; GGT, gamma-glutamyltransferase; IBD, inflammatory bowel disease; PSC, primary sclerosing cholangitis; PT, prothrombin time; UDCA, ursodeoxycholic acid; xULN, times upper limit of normal; VCTE, vibration-controlled transient elastography

### Baseline Pd-cT1 in healthy volunteers

**Fig. 4:**
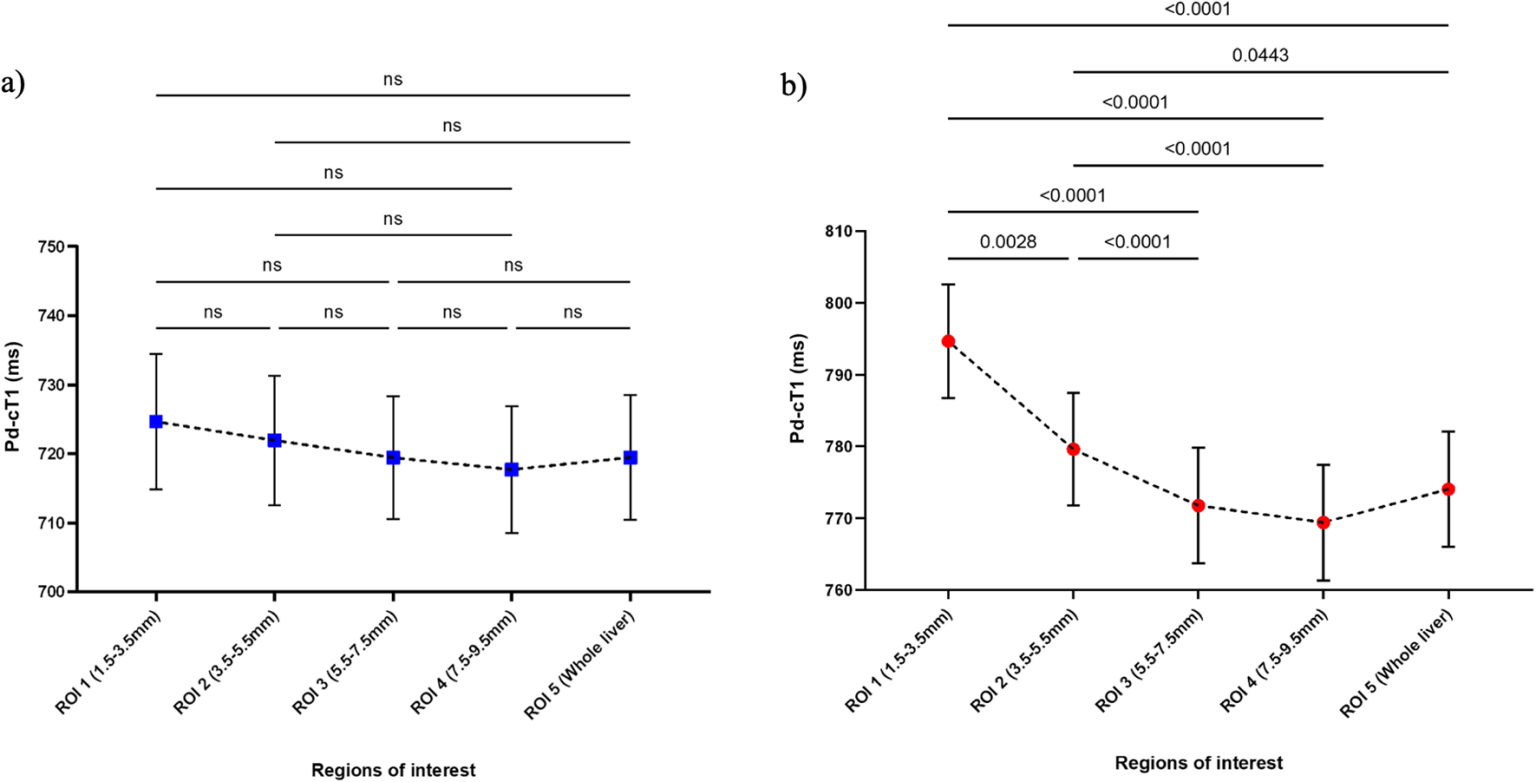
Mean ± standard error Pd-cT1 at fixed radial distances surrounding the bile ducts in **a**) in 20 healthy controls and **b**) 72 participants with PSC. ANOVA with Tukey’s multiple comparisons tests showing higher values in **a**) ROI 1 (1.5–3.5 mm) and ROI 2 (3.5–5.5 mm) compared to other periductal ROIs and whole liver mean. Only significant pairwise comparisons are depicted in a. ns, not significant

The mean Pd-cT1 in healthy volunteers were similar across all ROIs (*p* = 0.3661), with ROI 1 (1.5–3.5 mm): 725 ± 44ms; ROI 2 (3.5–5.5 mm): 722 ± 42ms; ROI 3 (5.5–7.5 mm): 719 ± 40ms; ROI 4 (7.5–9.5 mm): 718 ± 41ms; and ROI 5 (whole liver): 720 ± 41ms, (Fig. 4a).

### Baseline Pd-cT1 in PSC

In participants with PSC, the mean Pd-cT1 within the ring shaped ROIs were ROI 1 (1.5–3.5 mm): 795 ± 67ms; ROI 2 (3.5–5.5 mm): 780 ± 67ms; ROI 3 (5.5–7.5 mm): 772 ± 68ms; ROI 4 (7.5–9.5 mm): 769 ± 68ms; and ROI 5 (whole liver): 774 ± 68ms. In the post-hoc multiple comparisons tests, the mean Pd-cT1 in ROI 1 and 2 were higher compared to the other regions (*p* < 0.0001) but there were no significant differences between ROIs greater than 5.5 mm radius from the bile duct wall and the whole liver mean cT1 (Fig. 4b). The difference persisted (*p* < 0.0001) regardless of whether bile ducts intersected at less than 30° angle, between 30–60° angle, or between 60–90° angle to slice normal, (Figure S4). Mean Pd-cT1 in ROI 1 showed a trend of progressive increase with increasing oblique angle intersection of bile ducts.

### Baseline risk stratification

Whole liver cT1 and ROI1-4 Pd-cT1 showed positive correlations with LS (*r* = 0.28–0.36 *p* < 0.05) and was higher in those with LS > 9.6 kPa (799-833ms) compared to those with LS ≤ 9.6 kPa (749-781ms; *p* < 0.01), with increasing cT1 towards the ducts. Whole liver cT1 and ROI1-4 Pd-cT1 had AUC of 0.73–0.74 (95% CI 0.59–0.88), to differentiate the high-risk group from the low-risk group (Table S5). No correlation was found between cT1 and ELF, but participants with ELF > 9.8 had higher ROI1-3 Pd-cT1 compared to those with ELF ≤ 9.8 (*p* < 0.05) with AUC = 0.65–0.68, 95% CI 0.50–0.83 to discriminate high risk from low risk. Whole liver cT1 and ROI1-4 Pd-cT1 was higher in participants with AOM > 2 compared to those with AOM ≤ 2 (*p* < 0.01), and had an AUC of 0.70–0.72 (95% CI 0.57–0.86) to discriminate high-risk from low-risk group. There was no association of whole liver cT1 or Pd-cT1 with high risk stratified by the revised Mayo Risk Score > 0, 2.2x or 1.5x ULN ALP, Anali score, ISSS, presence of extrahepatic disease, hepatic dysmorphy, cirrhosis, portal hypertension or dominant strictures.

### Long term follow-up

**Fig. 5:**
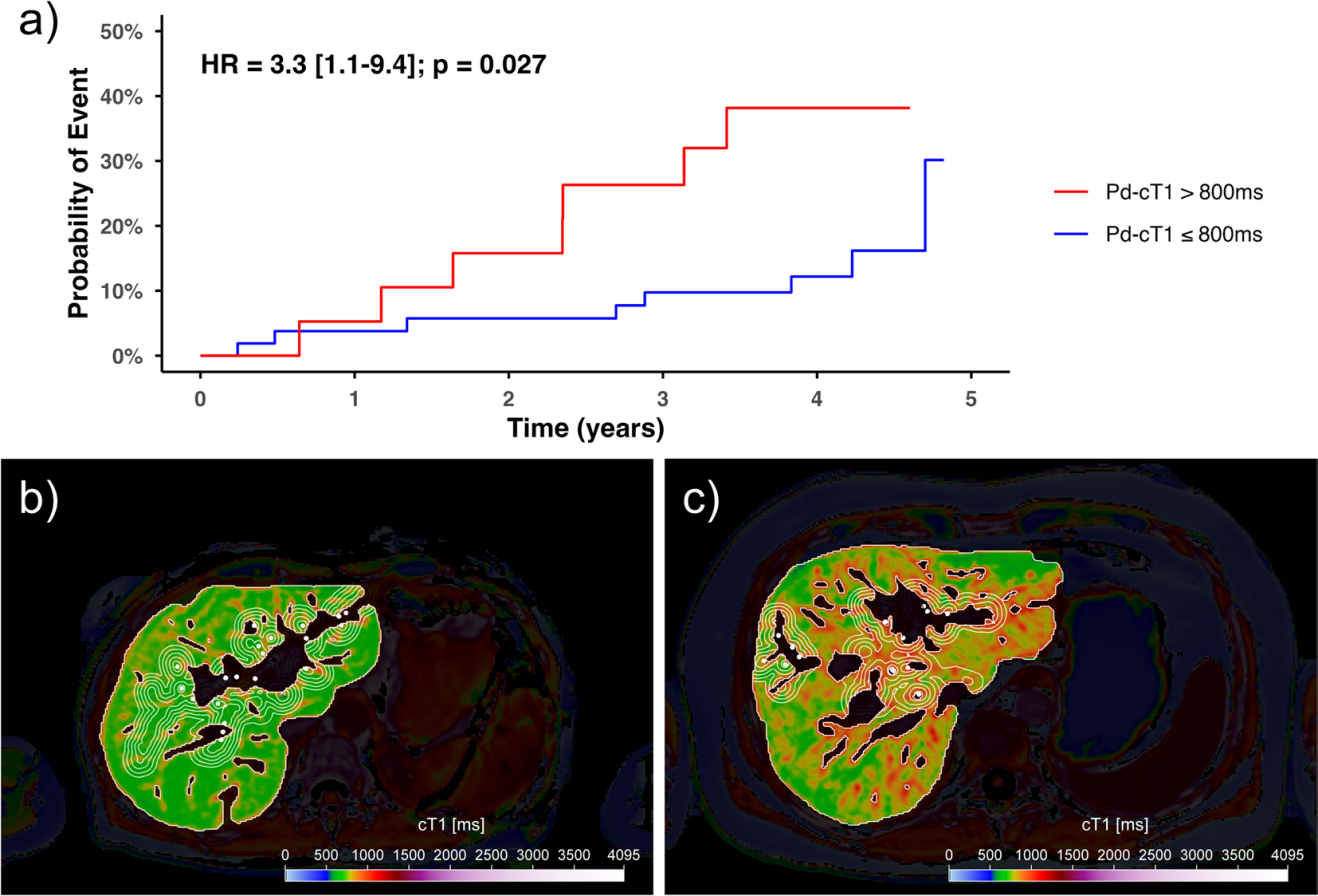
(**a**) Cumulative event probability curve and hazard ratio (HR) for elevated (> 800ms) vs. non-elevated (≤ 800ms) Pd-cT1 (ROI3; 5.5–7.5 mm) predicting adverse outcomes composite: cholangitis, hepatic decompensation, liver transplantation and all-cause mortality. Representative images of patients (**b**) without clinical events (whole liver median cT1: 726ms, Pd-cT1 ROI1: 742ms, Pd-cT1 ROI2: 746ms, Pd-cT1 ROI3: 742ms, Pd-cT1 ROI4: 737ms) versus (**c**) with clinical event mortality (whole liver median cT1: 775ms, Pd-cT1 ROI1: 853ms, Pd-cT1 ROI2: 828ms, Pd-cT1 ROI3: 818ms, Pd-cT1 ROI4: 806ms)

After a median follow-up of 49 months (range: 14–66), there were 46 adverse events in 24 of the total 72 participants, out of which 11 experienced more than one event. Events recorded included development of new dominant stricture (DS) (*n* = 8), the need for ERCP (*n* = 13), cholangitis (*n* = 10), gallbladder cancer (*n* = 1), hepatic decompensation (*n* = 8), liver transplantation (*n* = 3), and all-cause mortality (*n* = 3). Elevated (> 800ms) whole liver cT1 or Pd-cT1 were not predictive of the primary outcome transplant-free survival. However, elevated Pd-cT1 in ROI3 or ROI4 were predictive (HR: 3.22 [1.12–9.22] and 3.28 [1.14–9.40, respectively; *p* < 0.05) of the secondary outcome, which combines cholangitis, hepatic decompensation, liver transplantation and all-cause mortality (Fig. 5; Table S6). For prediction of the secondary outcomes, the optimal thresholds for ROI3-4 was 808ms and 798ms respectively, while slightly higher for ROI1-2 (822ms and 823ms, respectively). The sex- and age-adjusted HR for elevated Pd-cT1 (ROI4) to predict the secondary composite outcome by multivariable Cox proportional model was 4.07 [1.39–11.94]. The HRs stayed significant after further adjusting for LSM, ELF and ALP independently (HR: 3.59 [1.05–12.29, 3.70 [1.26–10.88] and 4.13 [1.39–12.23, respectively), while adjusting for AOM reduced the HR to 2.81 [0.81–9.70]. Elevated Pd-cT1 did not show significant prediction for biliary complications as a composite endpoint (new DS, need for ERCP, cholangitis and gallbladder cancer).

### Temporal change in Pd-cT1 in PSC

**Fig. 6:**
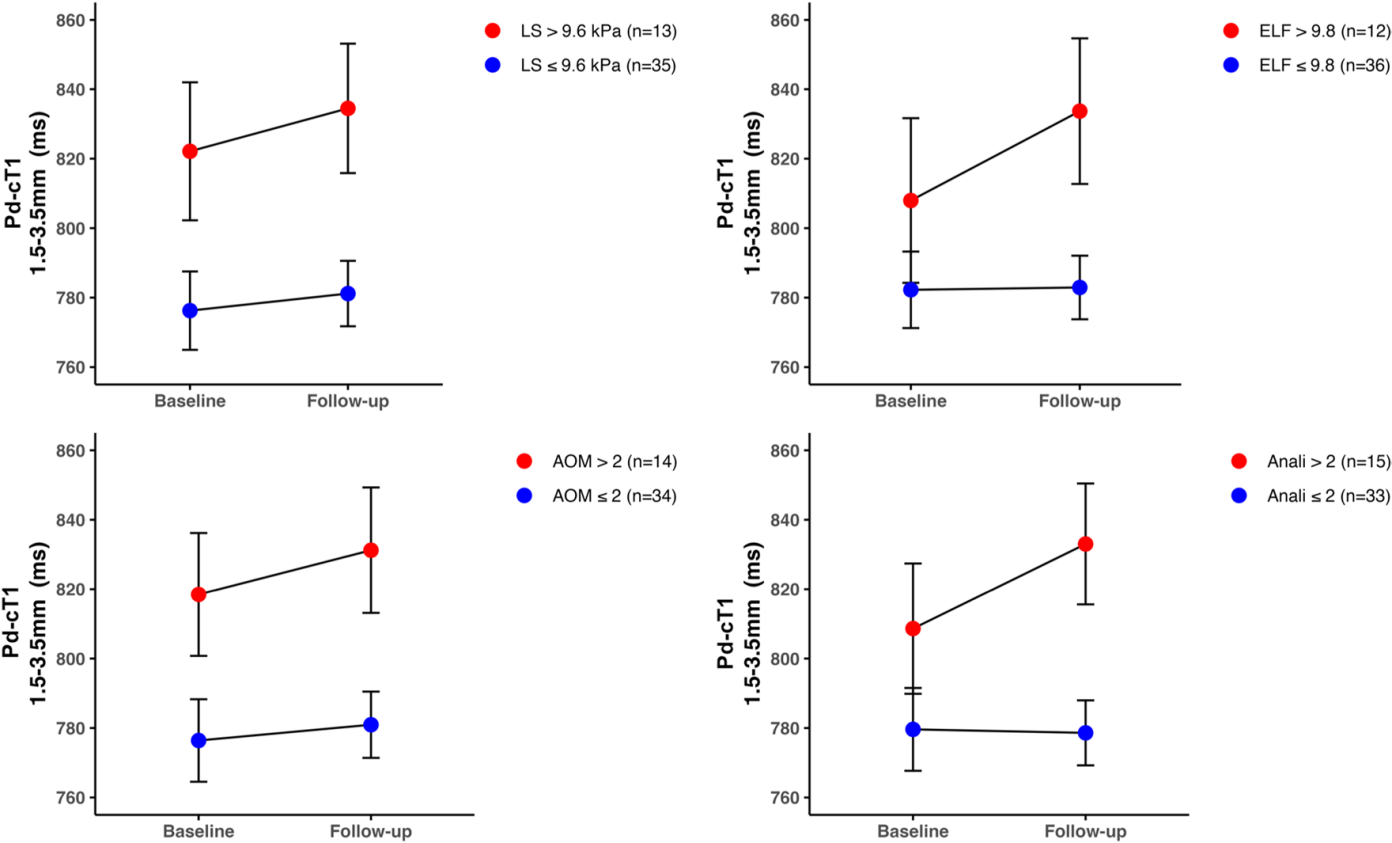
Paired mean Pd-cT1 in ROI 1 (1.5–3.5 mm) between baseline and follow-up study visits in high-risk (red) and low-risk (blue) groups in 48 participants with PSC stratified by baseline surrogate markers. **a**) LS > 9.6 kPa versus LS ≤ 9.6kPa; **b**) ELF > 9.8 versus ELF ≤ 9.8; **c**) AOM > 2 versus AOM ≤ 2; **d**) Anali > 2 versus Anali ≤ 2. There were no significant interactions between time point and baseline risk categorisation in the mixed-effects ANOVA models. AOM, Amsterdam-Oxford Model; LS; transient elastography liver stiffness; ELF, enhanced liver fibrosis score

The median interval between baseline and follow-up visit was 417 (IQR: 317–495) days. Paired within-subject characteristics of the 48 participants with PSC included in the follow-up analysis is summarised in Table S6. Similar to baseline analysis, a gradient was observed at follow-up visit with higher mean Pd-cT1 in ROIs 1 and 2 compared to ROIs 3–5 (ROI 1: 796 ± 63ms; ROI 2: 777 ± 63ms; ROI 3: 768 ± 64ms; ROI 4: 767 ± 63ms; and ROI 5: 759 ± 59ms; *p* < 0.0001). Furthermore, participants with higher risk scores at baseline had higher mean ROI1 Pd-cT1 at both timepoints compared to those with less severe disease at baseline (ANOVA models of LS [*p* = 0.014, ELF [*p* = 0.038, AOM [*p* = 0.019] and Anali [*p* = 0.032]), Table S8 and Fig. 6. On a cohort and risk-group level, there was no difference in Pd-cT1 between baseline and follow-up (Table S8) and the change in mean Pd-cT1 between the visits could not discriminate the high-risk from the low-risk groups (Table S9).

## Discussion

This work is the first to propose a method for objectively quantifying periductal abnormalities in PSC using a combination of two quantitative MRI techniques without the use of intravenous contrast agents. The key findings were that (1) participants with PSC had higher Pd-cT1 within 5.5 mm of the bile ducts compared to the overall liver parenchyma; (2) elevated Pd-cT1 was associated with measures of disease severity; and (3) elevated Pd-cT1 was predictive of adverse events.

The gradient of increasing Pd-cT1 closer to the bile ducts in PSC was not seen in healthy controls, suggesting that the elevated Pd-cT1 is disease-driven. Heterogeneity in cT1 across the liver parenchyma has previously been described in PSC, while lower cT1 heterogeneity was reported in other chronic liver diseases [45]. Abnormalities in bile ducts quantified by MRCP predicted elevated cT1 heterogeneity in children and young adults with PSC, supporting our findings of regional cT1 elevation in this cohort.

Elevated cT1 may reflect both fibrosis and inflammation in the liver parenchyma. This cohort consisted of stable, ambulatory patients without clinical or biochemical evidence of acute inflammation, and Pd-cT1 correlated with liver stiffness but not with serum ALT, AST or ALP. The association of Pd-cT1 with non-invasive fibrosis measures, rather than biochemical markers of liver disease activity, suggests that Pd-cT1 captures fibro-inflammatory remodeling of the periductal parenchyma; as this signal extends further from the bile ducts, it may reflect a transition toward more diffuse tissue involvement, which could be relevant to subsequent ductal complications such as stricture formation. In previous studies in children and young adults with autoimmune liver diseases, including autoimmune hepatitis, autoimmune sclerosing cholangitis (ASC) and PSC, whole liver median cT1 had a stronger association with inflammation, while the heterogeneity measure of cT1 (cT1 interquartile range [IQR]) was associated with fibrosis stage on histology [45, 46] and not correlated with biochemical markers of liver inflammation [45]. Studies using contrast-enhanced MRI, showed that early and delayed peribiliary hyperenhancement were linked to inflammatory and fibrotic processes, respectively [6, 7]. However, those methods rely on contrast and subjective interpretation, whereas Pd-cT1 avoids contrast administration for a more patient-centred and scalable approach and offers a quantitative alternative for evaluating periductal abnormalities in PSC. Objective and quantitative metrics enable longitudinal monitoring and assessment of treatment response, supporting potential use in clinical trials and equitable care. As Pd-cT1 builds on standardized cT1 mapping and 3D MRCP-based biliary modelling that are harmonized across vendors and field strengths and demonstrated low inter-user variability, it offers improved reproducibility over qualitative MRI, although future studies should formally evaluate inter-operator repeatability.

The risk group analyses suggest that Pd-cT1 can better risk stratify patients with PSC, compared to the assessment of whole liver parenchyma. The high risk groups stratified by fibrosis markers (liver stiffness and ELF) had higher cT1 than the low risk groups and, particularly in high risk by ELF, Pd-cT1 in the regions 1.5–7.5 mm from the ducts were elevated where whole liver cT1 was not. The Pd-cT1 closest to the bile ducts was also higher in participants with PSC classified as high-risk based on the AOM risk score. Qualitative radiological reads of biliary or parenchymal features and scores for risk assessment in the PSC cohort showed no association with whole liver nor Pd-cT1, which may emphasize the sensitivity of quantitative MRI for periductal fibrosis and indicate a lack of signal utilizing qualitative MRI assessments alone.

Elevated (> 800ms) Pd-cT1 at 5.5–9.5 mm from the ducts could predict events of cholangitis, hepatic decompensation, liver transplant or all-cause mortality in large-duct PSC. Pd-cT1 retained independent prognostic value beyond current serum and parenchymal markers of disease severity (LSM, ELF and ALP). The attenuation of the association after adjustment for the prognostic risk score AOM may suggest overlapping prognostic information, or an over-adjustment due to the composite nature of AOM. The slightly higher optimal threshold for ROI1-2 than ROI3-4 to reach predictive power for the same endpoint aligns with the evidence of higher Pd-cT1 closer to the ducts in PSC. This may suggest that when PSC disease activity centred around the ducts increase, and expands above normal levels further from the ducts, the risk of adverse events increase. The optimal thresholds for Pd-cT1 to predict outcomes in this cohort of patients with large-duct PSC were found to be similar to the well established upper limit of normal cT1 > 800ms. Although validation in a larger multi-centre dataset is needed, these results on prognostication of Pd-cT1 in PSC adds to previous evidence demonstrating association of cT1 with liver- and cardiovascular related events in population studies, and quantitative biliary tree metrics with adverse events in PSC [20, 22, 47–49].

Beyond the cross sectional evaluation context of the study, monitoring of Pd-cT1 over time showed that participants maintained a stable Pd-cT1 at the one year follow-up, with a trend of increasing Pd-cT1 in the high-risk participants. PSC is a slowly progressing disease and all the included parenchymal biomarkers, serum markers and risk scores showed no significant change over the follow-up period. Quantitative MRCP metrics have shown utility for monitoring patients with PSC over time, as it demonstrated changes in the biliary tree after one year that were not detected by biochemical markers [27]. Furthermore, worsening in cholangiopathy quantified by MRCP was associated with increasing liver stiffness in patients with PSC/ASC [50]. These findings warrant further studies on longitudinal changes in Pd-cT1 following intervention, to demonstrate utility in disease monitoring and its potential as an endpoint in antifibrotic drug trials for PSC.

Early stages of PSC can be difficult to detect with liver biochemistry or qualitative MRCP [51, 52, but studies using contrast enhanced MRI found signs of peribiliary enhancement in these early phenotype PSC [53]. This suggest that periductal disease activity may be present in PSC before bile duct irregularities are seen, and presents an avenue for future studies in early stage disease, to further investigate the utility of Pd-cT1. In addition, longitudinal investigations assessing the spatial trajectory of Pd-cT1 abnormalities may enable prediction of the anatomical sites of future disease progression, such as stricture formation or cholangitic episodes. If validated, this would support the use of Pd-cT1 not only as a biomarker of current disease severity but also as a spatially-resolved predictor of future ductal injury, with potential applications in targeted monitoring and preemptive intervention.

Our study has several strengths, like the prospective recruitment of participants and protocolised follow-up after 1 year. However, limitations must also be acknowledged. The rate of follow-up was lower that we would have liked, as many participants chose not to return for repeat assessment due to the COVID-19 pandemic. Furthermore, only healthy controls and individuals with PSC were included limiting any comparison with other liver diseases. Whilst none of the participants with PSC had recent cholangitis at study recruitment, the absence of histological comparison makes it difficult to conclude the relative contribution of periductal fibrosis and subclinical inflammation to elevated Pd-cT1. The absence of a ‘gold standard’ comparator for Pd-cT1 and the single-center design could be addressed in future multi-center, multi-vendor studies anchored in histology. MRCP resolution allows delineation of the biliary tree up to segmental ducts and Pd-cT1 is therefore limited to quantification around larger ducts. Depiction of smaller bile ducts including the septal, interlobular, and bile ductules are not possible as their diameter is less than 400µm [54, and these are the bile ducts typically sampled by liver biopsies. Despite strategies to mitigate partial-volume effects (distance thresholding and angle-aware exclusion), residual bile-signal contamination near ducts cannot be fully excluded.

## Conclusions

In conclusion, in this proof of concept study a novel method to quantitatively assess periductal abnormalities in PSC using a combination of noninvasive biliary and parenchymal quantitative MRI techniques was developed. Mean Pd-cT1 in regions closer to the bile ducts were observed to be higher than regions further away in PSC, but remained constant in healthy controls. Participants classified as high risk based on advanced fibrosis tests and PSC risk scores showed higher and increasing Pd-cT1 compared to low risk groups. Additionally, elevated Pd-cT1 was prognostic of adverse events. This study proposes a non-invasive, objective biomarker to assess fibro-inflammation in the periductal parenchyma in PSC. Once validated for technical stability and anchored to histology, Pd-cT1 could have the potential to serve as a monitoring biomarker and a clinical trial endpoint in high-risk PSC.

## Supplementary Information

Below is the link to the electronic supplementary material.

Supplementary Material 1

## Data Availability

The datasets used and/or analysed during the current study are available from the corresponding author on reasonable request.

## Abbreviations

ALP: Alkaline phosphatase
ALT: Alanine aminotransferase
AOM: Amsterdam-Oxford Model
ASC: Autoimmune sclerosing cholangitis
AST: Aspartate aminotransferase
AUC: Area under the receiving operator curve
cT1: Iron-corrected T1
DS: Dominant strictures
EASL: European Association for Study of the Liver
ELF: Enhanced Liver Fibrosis
GGT: Gamma-glutamyl transferase
HR: Hazard Ratio
HV: Healthy volunteer
IBD: Inflammatory bowel disease
IQR: Interquartile range
LS: Liver stiffness
MRCP: Magnetic resonance cholangiopancreatography
MRE: Magnetic resonance elastography
MRI: Magnetic resonance imaging
ms: Milliseconds
Pd-cT1: Periductal cT1
PSC: Primary sclerosing cholangitis
PT: Prothrombin time
ROC: Receiver-operator curve
ROI: Region of interest
SD: Standard deviation
ULN: Upper limit of normal
