# Supplementary material for: Periductal iron-corrected T1 is a predictor of adverse outcomes in large-duct primary sclerosing cholangitis
Source: BMC Med Imaging. 2026 Mar 4;26:186. doi: 10.1186/s12880-026-02242-1 (PMC13067436; doi:10.1186/s12880-026-02242-1)
Supplement: Supplementary file 1 — Supplementary Material 1 [file 12880_2026_2242_MOESM1_ESM.docx]

**Supplementary Material**

**Periductal iron-corrected T1 is a predictor of adverse outcomes in large-duct primary sclerosing cholangitis**

# **Supplementary Methods**

In MRI, partial volume effects occur when the components contributing to signal intensity come from more than one source. In periductal cT_1_ (Pd-cT1) quantification, partial volume effects could potentially arise at voxels of bile duct boundary, which may contain signals from within the bile duct, accompanying smaller portal blood vessel, and surrounding liver parenchyma at the level of intersection. Given that the MRCP acquisition voxel size was 1.1 x 1.1 x 1.1mm, Pd-cT1 measurements below 1.5mm distance from the bile duct wall were excluded to minimise partial volume effect.

Oblique bile duct orientation with respect to the axial liver cT_1_ map can also give rise to partial volume effect as demonstrated in **Figure S1.** Increasing the angle of deviation from the slice normal brings the bile ducts proximity closer to the liver, and therefore increases the number of Pd-cT_1_ voxels that is averaged out to contain signals from both the liver and bile. Freely moving protons in free water have very long T_1_ relaxation time — approximately 4000ms at 3 T. Bile is composed predominantly of water (97-98%). Therefore, bile has the effect of increasing the cT_1_ in the periductal regions closer to the bile ducts.

To be able to address partial volume effect in regions where bile ducts traverse through 2D image slices at an oblique angle, angles between slice normals and biliary tree duct segments at intersection points were calculated, where slice normals were taken to have inferior-superior direction. Voronoi maps were calculated for each slice and each Voronoi region within was assigned angle calculated at closest corresponding intersection point (**Figure S2**). For the analysis, bile ducts were divided between those lesser than 30° angle, between 30–60° angle, and between 60–90° angle. Regions of interests were expanded and re-defined as ROI 1 (1.5–5.5mm), ROI 2 (5.5–9.5mm), and ROI 3 (whole liver) to avoid missing values in certain periductal distance groups that would restrict pairwise comparisons to be performed for the cohort.

# **Supplementary Figures**


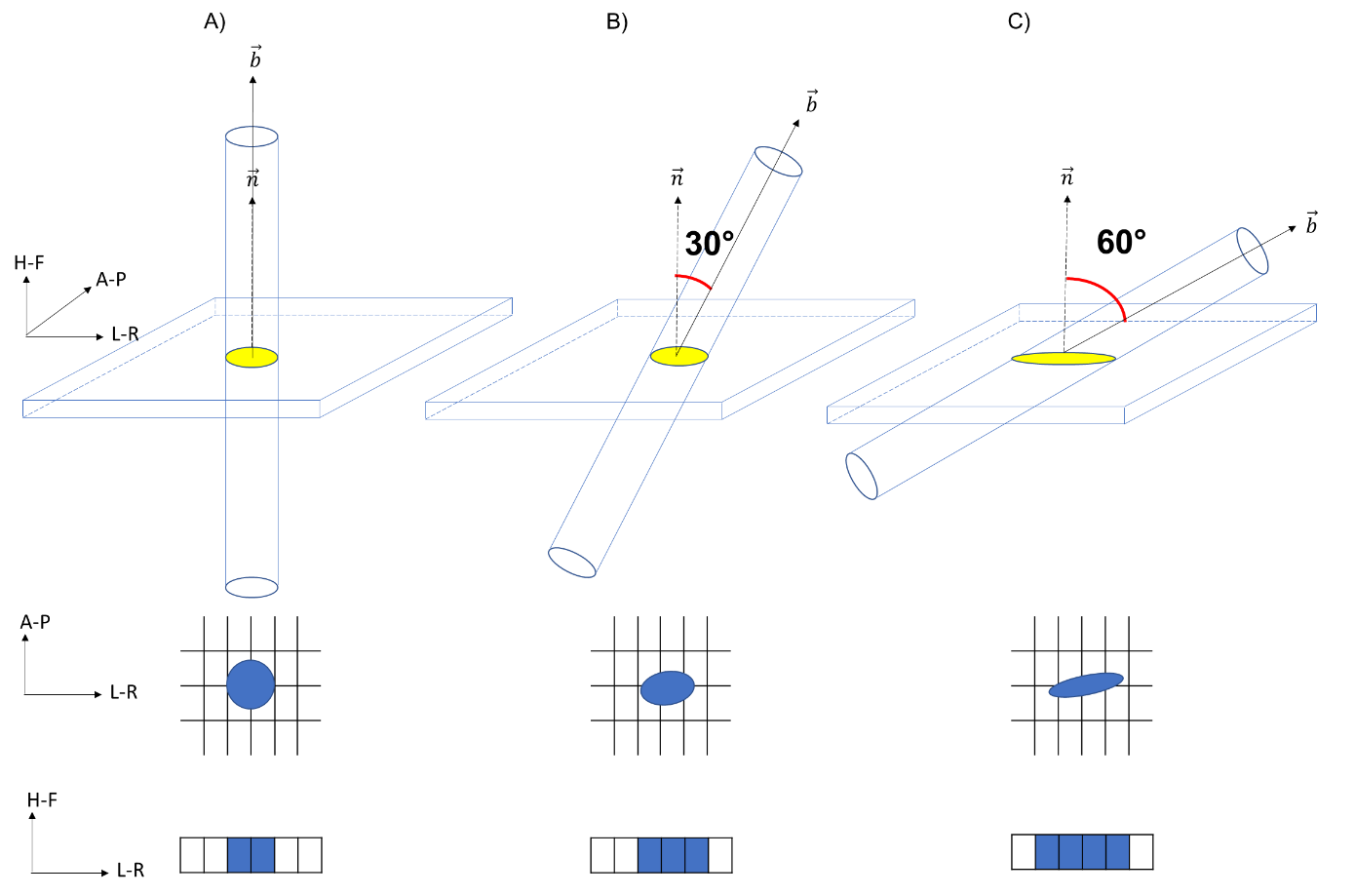


Figure S1. A schematic diagram representing partial volume effect exerted by a single bile duct intersecting an axial segmented liver cT_1_ slice at different angles. (A) 0°, (B) 30°, and (C) 60° to the slice normal represented by the dotted line (i.e., the angle between the normal vector of the imaging plane, $\vec{n}$ and the vector describing the orientation of the bile duct, $\vec{b}$).  Their respective proportion of partial volume voxels are represented on the axial (A-P and L-R axis, middle row) and coronal (H-F and L-R axis, bottom row) views. Abbreviation: A-P, anterior-posterior; L-R, left-right; H-F, head-feet.


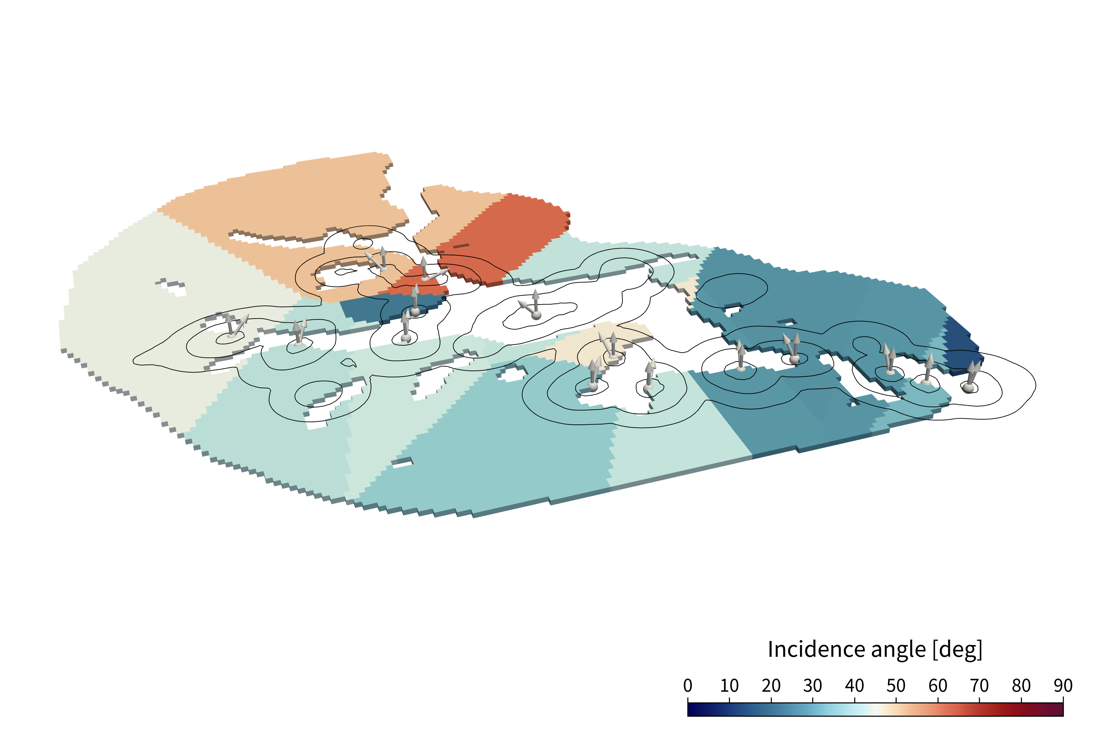


Figure S2. Voronoi map for a single axial liver slice where each voxel is assigned duct incident angle at closest intersection point. Arrow glyphs represent surface normals and duct directions at intersection points of biliary tree model with axial liver slice. Colour map represents duct incident angle to surface normal.


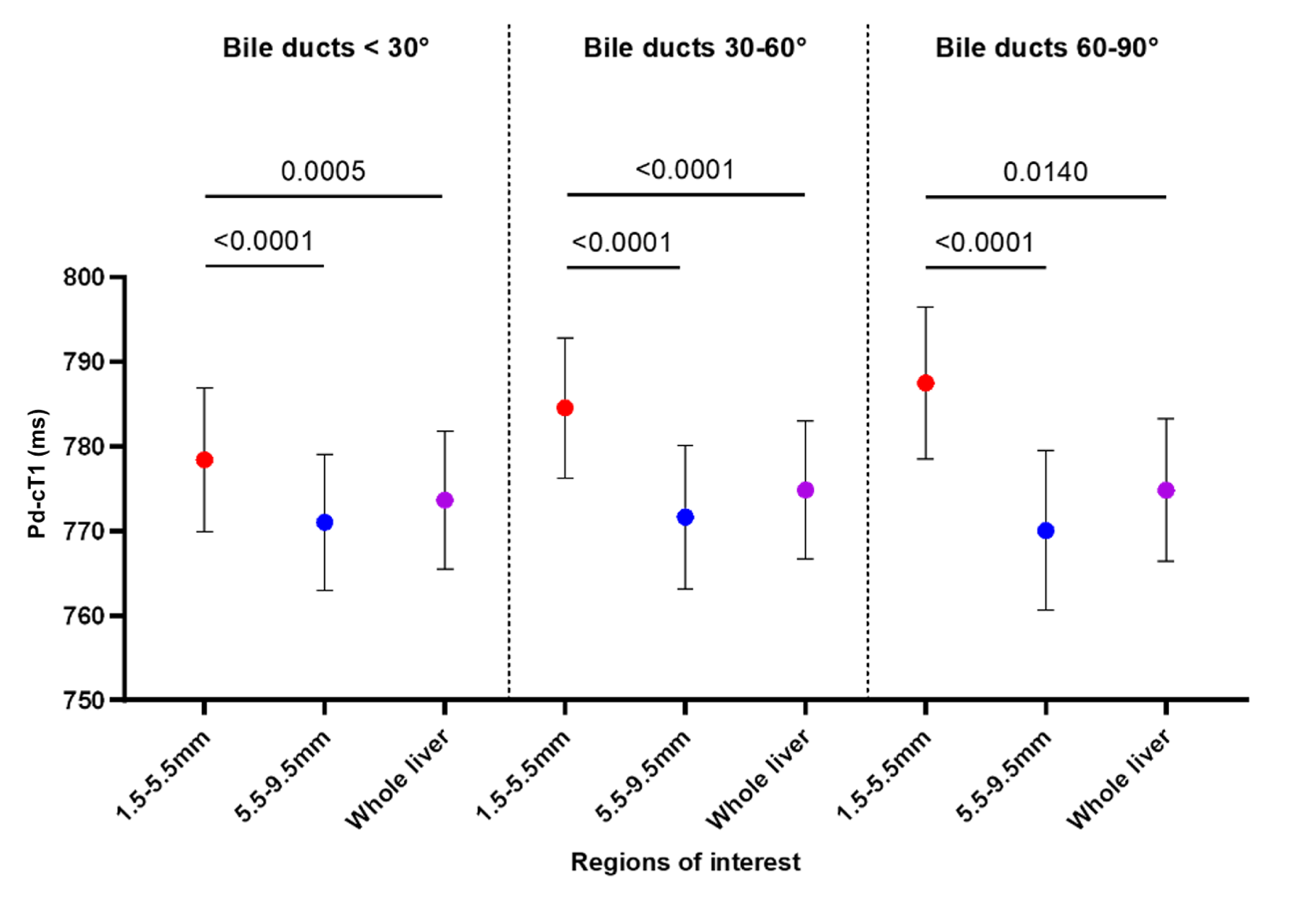


Figure S3. Mean ± standard error Pd-cT1 at fixed radial distances surrounding the bile ducts in 73 participants with PSC. b) expanded ROIs 1–2 (1.5–5.5mm) compared to ROI 3–4 (5.5–9.5mm) and whole liver mean corrected for partial volume effect from bile ducts intersecting at less than 30°, between 30–60° angle, or between 60–90° angle to slice normals. Only significant pairwise comparisons are depicted.

# **Supplementary Tables**

Table S1. MRI-MRCP acquisition parameters

| Parameter | MRCP | T1 | T2* | VIBE |
| --- | --- | --- | --- | --- |
| Sequence | 3D multi-shot fast/turbo spin echo | Shortened modified Look- Locker Inversion (shMOLLI) | Multi-echo gradient echo (GRE) | Double echo fast low angle shot (FLASH) |
| Plane | Coronal | Axial | Axial | Axial |
| Triggering | Free breathing, navigator based | Breath-hold, pulse or ECG-gated | Breath-hold, pulse or ECG-gated | Breath-hold, not triggered |
| Number of slices | 60 | 5 | 1 | 72 |
| Slice thickness, mm | 1.1 | 8 | 6 | 3 |
| Echo time (TE), ms | 604 | 1.05 | 1.23 with echo separation 1.23ms | 1.29, 2.52 |
| Repetition time (TR), ms | Breathing cycle | 2.43 | 22.2 | 3.97 |
| Acquisition matrix |  | 192 x 144 | 208 x 256 | 195 x 320 |
| FOV, mm | 256 x 256 | 440 x 330 (75% phase FOV) | 400 x 325 (81% phase FOV) | 309 x 380 |
| Flip angle (degrees) | 280 | 35 | 20 | 9 |
| iPAT factor | 120 | GRAPPA 2-fold with 24 reference lines | GRAPPA 2-fold with 35 reference lines | CAIPIRINHA 2-fold with 24 reference lines in both phase encoding and 3D |
| Voxel resolution, mm | 2 (GRAPPA, 24 reference lines, integrated mode) | 2.2 x 2.2 x 8.0  (interpolated to 1.1 x 1.1 x 8.0) | 3.1 x 3.1 x 6.0  (interpolated to 1.6 x 1.6 x 6.0) | 1.2 x 1.2 x 3.0 |
| Number of averages | 1.1 x 1.1 x 1.1 | 1.0 | 1.0 | 1.0 |
| Fat suppression | 1.4 | None | None | None |

Table S2: Modified Amsterdam cholangiographic classification of stricture severity.

| **Amsterdam score** | **Modified score** | **Description** |
| --- | --- | --- |
| ***Intrahepatic tree*** | | |
| 0 | 0 | No visible abnormalities |
| I | 1 | Ductular irregularities |
| I | 2 | Multiple calibre changes; minimal dilatation |
| II | 3 | Multiple strictures, saccular dilatations, decreased arborisation |
| III | 4 | Only central branches filled despite adequate filling pressure; severe pruning |
| ***Extrahepatic tree*** | | |
| 0 | 0 | No visible abnormalities |
| I | 1 | Slight irregularities of duct contour, no stricture |
| II | 2 | Segmental stricture |
| III | 3 | Stricture of almost entire length of duct |
| IV | 4 | Extremely irregular margins; diverticulum‐like outpouchings |

Table S3: Anali score classification.

| **Component** | **Score** | **Description** |
| --- | --- | --- |
| Intrahepatic bile duct dilatation* | 0 | 3mm or smaller |
|  | 1 | 4mm |
|  | 2 | 5mm or larger |
| Hepatic dysmorphy† | 0 | Absent |
|  | 1 | Present |
| Portal hypertension‡ | 0 | Absent |
|  | 1 | Present |
| *Intrahepatic duct with the largest diameter | | |
| †Significant atrophy of either the right or left hepatic lobe and/or marked lobulations of liver surface and/or increase of the caudate/right lobe ratio | | |
| ‡The presence of portosystemic shunts with or without splenomegaly | | |

Table S4. Baseline characteristics of 20 healthy controls.

|  | Healthy (n = 20) | |
| --- | --- | --- |
| Male (n (%)) | 13 | (65) |
| Age (years) | 35 | (31–39) |
| Total bilirubin (µmol/L) | 10 | (8–13) |
| ALT (IU/l) | 20 | (13–26) |
| AST (IU/l) | 22 | (19–24) |
| ALP (IU/l) | 58 | (51–70) |
| xULN ALP^†^ | 0.4 | (0.4–0.5) |
| GGT (IU/l) | 17 | (14–20) |
| Albumin (g/l) | 42 | (41–43) |
| Prothrombin time (s) | 10.5 | (10.5–11.0) |
| Platelet count (x 10^9^/l) | 256 | (234–270) |
| LS (kPa) | 4.7 | (4.1–5.3) |
| ELF | 8.6 | (8.0–8.9) |
| Continuous variables are expressed as median (interquartile range) and nominal variables as absolute number (percentage). ^†^ULN of ALP was 130 IU/L.  Abbreviations: ALP; alkaline phosphatase, ALT; alanine aminotransferase, AST; aspartate aminotransferase, ELF; enhanced liver fibrosis score; GGT; gamma-glutamyltransferase, LS; transient elastography liver stiffness, xULN; times upper limit of normal. | | |

Table S5: Baseline risk stratification of Pd-cT1, classified by other non-invasive markers of fibrosis or disease risk scores.

| Risk group stratification | ROI 1 (1.5–3.5mm) | ROI 2 (3.5–5.5mm) | ROI 3 (5.5–7.5mm) | ROI 4 (7.5–9.5mm) | Whole liver |
| --- | --- | --- | --- | --- | --- |
| Risk group by LS | | | | | |
| Correlation with LS | r=0.36; p=0.0018 | r=0.34; p=0.004 | r=0.33; p=0.0047 | r=0.33; p=0.0046 | r=0.28; p=0.019 |
| Mean cT1 (ms) in LS > 9.6 | 833±76 | 819±75 | 812±77 | 810±76 | 799±78 |
| Mean cT1 (ms) in LS ≤ 9.6 | 781±59 | 766±58 | 757±59 | 755±60 | 749±62 |
| Test difference in mean (p) | p=0.0026 | p=0.0017 | p=0.0019 | p=0.0017 | p=0.0036 |
| Test stratification (AUC) | 0.73 [0.59–0.88] | 0.74 [0.61–0.88] | 0.74 [0.6–0.88] | 0.74 [0.61–0.88] | 0.73 [0.59–0.87] |
| Risk group by ELF | | | | | |
| Correlation with ELF | r=0.12; p=0.31 | r=0.13; p=0.28 | r=0.11; p=0.37 | r=0.09; p=0.44 | r=0.08; p=0.48 |
| Mean cT1 (ms) in ELF > 9.8 | 824±80 | 806±82 | 798±84 | 795±83 | 787±83 |
| Mean cT1 (ms) in ELF ≤ 9.8 | 783±58 | 769±58 | 762±59 | 759±60 | 752±62 |
| Test difference in mean (p) | p=0.019 | p=0.034 | p=0.046 | p=0.057 | p=0.054 |
| Test stratification (AUC) | 0.68 [0.53–0.83] | 0.66 [0.51–0.82] | 0.65 [0.5–0.81] | 0.65 [0.49–0.8] | 0.65 [0.49–0.8] |
| Risk group by AOM | | | | | |
| Correlation with AOM | r=0; p=0.99 | r=0.01; p=0.91 | r=0.02; p=0.86 | r=0.02; p=0.9 | r=0.01; p=0.95 |
| Mean cT1 (ms) in AOM > 2 | 827±71 | 812±73 | 804±75 | 801±76 | 793±76 |
| Mean cT1 (ms) in AOM ≤ 2 | 780±60 | 766±59 | 757±60 | 755±61 | 748±63 |
| Test difference in mean (p) | p=0.0036 | p=0.0033 | p=0.0043 | p=0.0061 | p=0.0051 |
| Test stratification (AUC) | 0.72 [0.58–0.85] | 0.72 [0.58–0.86] | 0.71 [0.58–0.85] | 0.7 [0.57–0.84] | 0.71 [0.57–0.85] |
| Risk group by MRS | | | | | |
| Mean cT1 (ms) in MRS > 0 | 806±78 | 791±79 | 783±82 | 780±82 | 773±83 |
| Mean cT1 (ms) in MRS ≤ 0 | 780±47 | 766±44 | 758±43 | 756±44 | 748±47 |
| Test difference in mean (p) | p=0.12 | p=0.15 | p=0.18 | p=0.16 | p=0.18 |
| Risk group by serum markers | | | | | |
| Correlation with ALT | r=-0.14; p=0.25 | r=-0.15; p=0.21 | r=-0.16; p=0.17 | r=-0.18; p=0.13 | r=-0.18; p=0.13 |
| Correlation with AST | r=-0.01; p=0.92 | r=-0.02; p=0.85 | r=-0.04; p=0.72 | r=-0.06; p=0.64 | r=-0.08; p=0.52 |
| Correlation with ALP | r=0.1; p=0.42 | r=0.09; p=0.43 | r=0.08; p=0.52 | r=0.07; p=0.57 | r=0.06; p=0.63 |
| Test difference in mean cT1 ALP > 1.5xULN vs. ALP ≤ 1.5xULN | p=0.32 | p=0.23 | p=0.2 | p=0.24 | p=0.25 |
| Test difference in mean cT1 ALP > 2.2xULN vs. ALP ≤ 2.2xULN | p=0.62 | p=0.58 | p=0.68 | p=0.72 | p=0.69 |
| All correlations (r) refer to Pearson correlations, significant tests were done with t-test, mean presented as mean±SD and AUC with [95%CI]. ROI: periductal region of interest; LS: transient elastography liver stiffness; cT1: corrected T1; AUC: area under the receiving operator curve; ELF: enhanced liver fibrosis; AOM: Amsterdam-Oxford model; MRS: Mayo-risk score; ALT: alanine aminotransferase; AST aspartate aminotransferase; ALP: alkaline phosphatase; ULN: upper limit of normal. | | | | | |

Table S6: Hazard ratios (HR) for prediction of composite outcomes of adverse events by elevated Pd-cT1 and whole liver cT1 at upper limit of normal (cT1 > 800ms) and optimal thresholds.

| Composite outcomes | Threshold | ROI 1  (1.5–3.5mm) | ROI 2  (3.5–5.5mm) | ROI 3  (5.5–7.5mm) | ROI 4  (7.5–9.5mm) | Whole liver |
| --- | --- | --- | --- | --- | --- | --- |
| Primary outcome | | | | | | |
| All-cause mortality;  Liver transplantation | Elevated cT1  (> 800 ms) | 3.72 [0.67-20.55] | 2.98 [0.59-14.97] | 3.14 [0.63-15.73] | 3.19 [0.64-15.97] | 2.42 [0.43-13.47] |
|  | Optimal | cT1>792ms: 6.17 [0.71-53.5] | cT1>823ms: 4.45 [0.89-22.27] | cT1>814ms: **5.19 [1.02-26.34]*** | cT1>805ms: 3.61 [0.73-18.01] | cT1>818ms:  5.57 [0.93-33.42] |
| Secondary outcomes | | | | | | |
| All-cause mortality;  Liver transplantation;  Hepatic decompensation | Elevated cT1  (> 800 ms) | 2.06  [0.58-7.26] | 1.44  [0.36-5.83] | 2.65  [0.7-9.98] | 2.65  [0.7-9.98] | 1.31 [0.27-6.41] |
|  | Optimal | cT1>810ms 2.89 [0.77-10.89] | cT1>732ms:  HR NA^#^ | cT1>808ms 3.41 [0.91-12.86] | cT1>722ms:  HR NA^#^ | cT1>713ms:  HR NA^#^ |
| All-cause mortality;  Liver transplantation;  Hepatic decompensation; Cholangitis | Elevated cT1  (> 800 ms) | 2.19  [0.78-6.13] | 2.15  [0.74-6.21] | **3.25**  **[1.13-9.3]*** | **3.31**  **[1.15-9.48]*** | 2.66 [0.88-8.01] |
|  | Optimal | cT1>822ms: **3.22 [1.12-9.22]*** | cT1>822ms: **3.3 [1.14-9.57]*** | cT1>808ms: **4.31 [1.5-12.39]*** | cT1>798ms: **3.02 [1.05-8.66]*** | cT1>713ms:  HR NA^#^ |
| Exploratory outcomes | | | | | | |
| All-cause mortality;  Liver transplantation;  Cholangitis | Elevated cT1  (> 800 ms) | **3.48**  **[1.04-11.58]*** | 2.77  [0.89-8.61] | **4.31**  **[1.36-13.61]*** | **4.4**  **[1.39-13.93]*** | **3.32 [1.05-10.52]*** |
|  | Optimal | cT1>792ms: **5.99 [1.31-27.43]*** | cT1>823ms: **4.41 [1.42-13.72]*** | cT1>808ms: **5.76 [1.82-18.26]*** | cT1>798ms: **4.03 [1.28-12.74]*** | cT1>803ms: **3.63 [1.15-11.49]*** |
| Cholangitis | Elevated cT1  (> 800 ms) | 2.62 [0.74-9.33] | 1.86 [0.52-6.61] | 3.11 [0.9-10.78] | 3.19 [0.92-11.07] | 3.14 [0.88-11.21] |
|  | Optimal | cT1>792ms: **4.81 [1.02-22.72]*** | cT1>775ms: **4.91 [1.04-23.22]*** | cT1>757ms: **7.92 [1-62.62]*** | cT1>759ms: 7.4 [0.94-58.55] | cT1>720ms:  HR NA^#^ |
| Cholangitis;  ERCP;  GB cancer;  New DS | Elevated cT1  (> 800 ms) | 1.24  [0.52-2.96] | 1.1  [0.43-2.85] | 1.52  [0.61-3.78] | 1.57  [0.63-3.89] | 1.5 [0.55-4.13] |
|  | Optimal | cT1>756ms: 2.59 [0.76-8.82] | cT1>823ms: 1.77 [0.69-4.58] | cT1>808ms: 2.06 [0.83-5.13] | cT1>794ms: 1.6 [0.66-3.88] | cT1>715ms: 2.03 [0.6-6.89] |
| **p<*0.05  ^#^no HR could be calculated as all the events occurred in the high-risk group.  Reporting the hazard ratio (HR) with [95% confidence interval (CI)]. GB cancer: gallbladder cancer. ROI: region of interest. DS: dominant stricture. | | | | | | |

Table S7: Paired characteristics of the 48 participants included in the follow-up analysis

|  | PSC (n = 48) | | | |  | | |
| --- | --- | --- | --- | --- | --- | --- | --- |
|  | Visit 1 | | Visit 2 | | | p-value |  |
| *Demographics* |  |  |  |  |  |  |  |
| Male n (%) | 29 | (60) | 29 | (60) | | >0.99 |  |
| Age (years) | 44 | (33–59) | 45 | (34–59) | | 0.58 |  |
| *Laboratory parameters* |  |  |  |  | | |  |
| Total bilirubin (µmol/l) | 13 | (11–20) | 13 | (10–18) | | 0.86 |  |
| ALP (IU/l) | 144 | (97–231) | 126 | (107–224) | | 0.94 |  |
| ALT (IU/l) | 42 | (27–84) | 39 | (27–76) | | 0.85 |  |
| AST (IU/l) | 38 | (26–59) | 35 | (24–51) | | 0.38 |  |
| GGT (IU/l) | 119 | (49–308) | 98 | (49–273) | | 0.5 |  |
| Albumin (g/l) | 40 | (38–42) | 39 | (37–42) | | 0.52 |  |
| Prothrombin time (s) | 10.5 | (10.3–11.0) | 10.7 | (10.3–11.0) | | 0.56 |  |
| Platelet count (x 10^9^/l) | 256 | (200–297) | 243 | (194–297) | | 0.94 |  |
| cT1 |  |  |  |  | |  |  |
| Whole Liver | 765 | (724-797) | 755 | (722-791) | | 0.67 |  |
| ROI 1 (1.5-3.5mm) | 783 | (747-822) | 789 | (746-817) | | 0.5 |  |
| ROI 2 (3.5-5.5mm) | 767 | (734-811) | 776 | (732-818) | | 0.71 |  |
| ROI 3 (5.5-7.5mm) | 758 | (722-797) | 761 | (722-806) | | 0.73 |  |
| ROI 4 (7.5-9.5mm) | 759 | (724-800) | 760 | (724-806) | | 0.57 |  |
| *Clinical data* |  |  |  |  | | |  |
| PSC disease distribution |  |  |  |  | | >0.99 |  |
| Intrahepatic + extrahepatic | 27 | (56) | 27 | (56) | |  |  |
| Intrahepatic only | 21 | (44) | 21 | (44) | |  |  |
| On UDCA therapy | 25 | (52) | 25 | (52) | | >0.99 |  |
| IBD present | 35 | (73) | 35 | (73) | | >0.99 |  |
| IBD phenotype |  |  |  |  | | >0.99 |  |
| Ulcerative colitis | 24 | (67) | 24 | (67) | |  |  |
| Crohn’s | 6 | (17) | 6 | (17) | |  |  |
| Unspecified | 5 | (14) | 5 | (14) | |  |  |
| *Risk classifications* |  |  |  |  | | |  |
| xULN ALP | 1.6 | (1.3–2.0) | 1.5 | (1.2–1.8) | | >0.99 |  |
| VCTE liver stiffness (kPa) | 6.9 | (5.0–10.0) | 6.5 | (5.3–10.7) | | 0.91 |  |
| ELF score | 9.3 | (8.7–9.9) | 9.3 | (9.0–9.8) | | 0.76 |  |
| Amsterdam-Oxford Model score (AOM) | 1.6 | (1.3–2.0) | 1.5 | (1.2–1.8) | | 0.15 |  |
| Mayo Risk Score | 0.0 | (-0.2–0.4) | 0.1 | (-0.3–0.5) | | 0.64 |  |
| Continuous variables are expressed as median (interquartile range) and nominal variables as absolute number (percentage).  ALP, alkaline phosphatase; ALT, alanine aminotransferase; AST, aspartate aminotransferase; DS, dominant stricture; EHD, extrahepatic disease; ELF, enhanced liver fibrosis score; GGT, gamma-glutamyltransferase; IBD, inflammatory bowel disease; ISSS, modified Amsterdam intrahepatic stricture severity score; PT, prothrombin time; PSC, primary sclerosing cholangitis; UDCA, ursodeoxycholic acid; xULN, times upper limit of normal. | | | | |  | | |

Table S8: Summary of mixed-effects ANOVA models assessing the interaction of mean Pd-cT1 1.5–3.5mm with time point and risk categorisation stratified by non-invasive surrogate markers of disease severity.

|  | PSC (n=48) | | | |
| --- | --- | --- | --- | --- |
|  | Mean square | F-ratio (F) | p-value | Effect size (η_p_^2)^ |
| LS |  |  |  |  |
| Within-subject: Time point | 1415.1 | 1.4 | 0.241 | 0.030 |
| Time point x LS >9.6 (n=13)/≤9.6kPa (n=35) | 262.5 | 0.3 | 0.611 | 0.006 |
| Between-subject: LS >9.6 (n=13)/≤9.6kPa (n=35) | 46612.5 | 6.6 | **0.014** | 0.125 |
| ELF |  |  |  |  |
| Within-subject: Time point | 3134.4 | 3.3 | 0.076 | 0.067 |
| Time point x ELF >9.8 (n=12)/≤9.8 (n=36) | 2823.3 | 3.0 | 0.091 | 0.061 |
| Between-subject: ELF >9.8 (n=12)/≤9.8 (n=36) | 26311.4 | 4.5 | **0.038** | 0.071 |
| AOM |  |  |  |  |
| Within-subject: Time point | 1482.0 | 1.5 | 0.230 | 0.031 |
| Time point x AOM >2 (n=14)/≤2 (n=34) | 334.2 | 0.3 | 0.566 | 0.007 |
| Between-subject: AOM (n=14)/≤2 (n=34) | 42283.4 | 5.9 | **0.019** | 0.113 |
| MRS |  |  |  |  |
| Within-subject: Time point | 1189.4 | 1.2 | 0.282 | 0.025 |
| Time point x MRS >0 (n=23)/≤0 (n=25) | 172.8 | 0.2 | 0.680 | 0.004 |
| Between-subject: MRS (n=23)/≤0 (n=25) | 11840.6 | 1.5 | 0.226 | 0.032 |
| 1.5x ULN ALP |  |  |  |  |
| Within-subject: Time point | 2137.1 | 2.2 | 0.146 | 0.045 |
| Time point x ULN ALP >1.5x (n=15)/≤1.5x (n=33) | 1544.7 | 1.6 | 0.215 | 0.033 |
| Between-subject: ULN ALP >1.5x (n=15)/≤1.5x (n=33) | 7588.6 | 1.0 | 0.334 | 0.020 |
| Anali |  |  |  |  |
| Within-subject: Time point | 2822.0 | 3.0 | 0.089 | 0.061 |
| Time point x Anali >2 (n=15)/≤2 (n=33) | 3327.7 | 3.6 | 0.066 | 0.072 |
| Between-subject: Anali >2 (n=15)/≤2 (n=33) | 35889.7 | 4.9 | **0.032** | 0.096 |
| ISSS |  |  |  |  |
| Within-subject: Time point | 1153.9 | 1.1 | 0.289 | 0.024 |
| Time point x ISSS >2 (n=24)/≤2 (n=24) | 253.0 | 0.3 | 0.618 | 0.005 |
| Between-subject: ISSS (n=24)/≤2 (n=24) | 3028.1 | 0.4 | 0.543 | 0.008 |
| EHD |  |  |  |  |
| Within-subject: Time point | 1356.2 | 1.4 | 0.249 | 0.029 |
| Time point x EHD Yes (n=27)/No (n=21) | 624.7 | 0.6 | 0.432 | 0.013 |
| Between-subject: EHD Yes (n=27)/No (n=21) | 5386.6 | 0.7 | 0.416 | 0.014 |
| DS |  |  |  |  |
| Within-subject: Time point | 682.9 | 0.7 | 0.413 | 0.015 |
| Time point x DS Yes (n=16)/No (n=32) | 312.7 | 0.3 | 0.579 | 0.007 |
| Between-subject: DS Yes (n=16)/No (n=32) | 9810.3 | 1.2 | 0.271 | 0.026 |
| Note: ALP threshold 2.2x ULN was not examined as it violated the Box’s test of equality of covariance matrices. | | | | |
| MRS, Mayo Risk Score; AOM, Amsterdam-Oxford Model; ALP; alkaline phosphatase; LS; transient elastography liver stiffness; ELF, enhanced liver fibrosis score; ISSS, modified Amsterdam intrahepatic stricture severity score; EHD, presence of extrahepatic disease; DS, presence of dominant stricture. | | | | |

Table S9: Performance of mean Pd-cT1 1.5–3.5mm at baseline and change from baseline to discriminate high-risk vs low-risk groups stratified by non-invasive surrogate markers.

|  |  | Pd-cT1 1.5–3.5mm (n=48) | | | |
| --- | --- | --- | --- | --- | --- |
|  |  | Visit 1 | | Δ Visit 1–2 | |
|  |  | AUC (95% CI) | p-value | AUC (95% CI) | p-value |
|  |  |  |  |  |  |
| LS >9.6kPa vs ≤9.6kPa |  | 0.71 (0.52-0.89) | **0.0301** | 0.54 (0.36-0.72) | 0.6847 |
| ELF >9.8 vs ≤9.8 |  | 0.6 (0.38-0.81) | 0.3059 | 0.67 (0.5-0.85) | 0.0741 |
| AOM >2 vs AOM ≤2 |  | 0.7 (0.54-0.87) | **0.0278** | 0.53 (0.34-0.72) | 0.7855 |
| MRS > 0 vs MRS ≤0 |  | 0.56 (0.39-0.73) | 0.4893 | 0.49 (0.32-0.66) | 0.9096 |
| ULN ALP >2.2x vs ≤2.2x |  | 0.52 (0.17-0.87) | 0.8516 | 0.44 (0.22-0.66) | 0.6401 |
| ULN ALP >1.5x vs ≤1.5x |  | 0.56 (0.37-0.75) | 0.4975 | 0.42 (0.25-0.6) | 0.4042 |
| Anali >2 vs Anali ≤2 |  | 0.65 (0.47-0.83) | 0.0975 | 0.64 (0.45-0.82) | 0.1276 |
| ISSS >2 vs ≤2 |  | 0.55 (0.38-0.72) | 0.5362 | 0.48 (0.32-0.65) | 0.8528 |
| EHD present vs absent |  | 0.54 (0.38-0.71) | 0.6253 | 0.58 (0.41-0.75) | 0.3443 |
| DS present vs absent |  | 0.48 (0.29-0.67) | 0.8269 | 0.54 (0.35-0.74) | 0.6150 |
| ALP; alkaline phosphatase; AOM, Amsterdam-Oxford Model; DS, presence of dominant stricture; EHD, presence of extrahepatic disease; ELF, enhanced liver fibrosis score; ISSS, modified Amsterdam intrahepatic stricture severity score; LS; transient elastography liver stiffness; MRS, Mayo Risk Score. | | | | | |
